# Three-dimensional tooth surface texture analysis on stall-fed and wild boars (*Sus scrofa*)

**DOI:** 10.1371/journal.pone.0204719

**Published:** 2018-10-23

**Authors:** Eisuke Yamada, Mugino O. Kubo, Tai Kubo, Naoki Kohno

**Affiliations:** 1 Department of Evolutionary Studies of Biosystems, School of Advanced Sciences, SOKENDAI (The Graduate University for Advanced Studies), Hayama, Kanagawa, Japan; 2 Yamanashi Prefectural Museum, Fuefuki, Yamanashi, Japan; 3 The University Museum, The University of Tokyo, Bunkyo-ku, Tokyo, Japan; 4 Department of Natural Environmental Studies, Graduate School of Frontier Sciences, The University of Tokyo, Kashiwa, Chiba, Japan; 5 Department of Geology and Paleontology, National Museum of Nature and Science, Tsukuba, Ibaraki, Japan; 6 Graduate School of Life and Environmental Sciences, University of Tsukuba, Tsukuba, Ibaraki, Japan; Monash University, AUSTRALIA

## Abstract

Categorizing the archaeological remains of *Sus scrofa* as domesticated “pigs” or wild “boars” is often difficult because of their morphological and genetic similarities. For this purpose, we tested whether feeding ecological change of *S*. *scrofa* that accompanied their domestication can be detected based on the three-dimensional texture created on the tooth enamel surface by mastication. We scanned the lower tooth surface of one wild and one stall-fed populations of modern *S*. *s*. *leucomystax* and one wild population of *S*. *s*. *riukiuanus* by using a confocal laser microscope. The average body weight of *S*. *s*. *leucomystax* is twice as heavier as that of *S*. *s*. *riukiuanus*. The textures were quantified using the industrial “roughness” standard, ISO 25178, to prevent inter-observer errors and to distinguish small differences that were difficult to detect by two dimensional image observation. The values of parameters related to height and volume were significantly larger in the stall-fed population. Twenty parameters differed significantly between the stall-fed and wild population of *S*. *s*. *leucomystax*, which indicated that the feeding ecological difference affected the ISO parameters of the two boar populations. Six parameters also differed between the wild populations of *S*. *s*. *leucomystax* and *S*. *s*. *riukiuanus*. Surprisingly, no parameter differed between the populations of stall-fed *S*. *s*. *leucomystax* and wild *S*. *s*. *riukiuanus*. Consumption of hard nuts and/or agricultural fruits and crops by the wild population of *S*. *s*. *riukiuanus* may have produced a tooth surface texture similar to that of the stall-fed population of *S*. *s*. *leucomystax*. Further analysis of *S*. *s*. *riukiuanus* with a known diet is necessary to conclude whether ISO parameters reflect the dietary transition accompanying the domestication of *Sus* (e.g., wild, semi-domestic, and domestic). Until then, caution is needed in discriminating domesticated populations from wild populations that mainly feed on hard objects.

## Introduction

Distinguishing domestic pig from wild boar is often difficult because they are the same species, *Sus scrofa*. Their body sizes and shapes vary corresponding to the climate and nutrition conditions. As a result, the morphology of pigs and boars often overlaps. Genetic characteristics are also unsuitable for distinguishing them because they can interbreed easily. Therefore, dichotomous approaches for the identification of zooarchaeological remains as pig or boar are insufficient to describe the complex domestication process of *S*. *scrofa*. Instead, the life style of each individual (i.e., whether they were reared, wild, or feral etc.) can be the key to understanding the continuous domestication process [1].

For decades, the examination of microwear using scanning electron microscopy (SEM) has been used for the dietary reconstruction of fossil species, including those of human ancestors [2]. Furthermore, Ward and Mainland [3] reported that dental microwear is effective in distinguishing stall-fed pigs from free-range paddocked boar. The former showed a low frequency of scratches and a high frequency of pits, which reflect the consumption of soft pellets, whereas the latter showed relatively dense microwear features with a high frequency of scratches, which was caused by soil ingestion during rooting. Based on this report, several studies have already applied microwear analysis to zooarchaeological remains for inferring the raising conditions of *Sus* to determine whether they were fed [4–6]. Assessing dental microwear is particularly efficient for determining the diet at the time of slaughter or hunting of *S*. *scrofa* because it reflects the diet and foraging behavior of the animal a few days prior to death [7, 8].

To overcome the disadvantages of microwear analysis based on two-dimensional images (2D), which are prone to inconsistency in scoring definitions [9] and inter-observer errors [10], three-dimensional (3D) dental microwear texture analysis with an automated quantification of “roughness” parameters has recently been developed. For the calculation of roughness, two standards have been proposed: scale sensitive fractal analysis [11, 12] and surface texture analysis (STA [13, 14]) using the surface roughness standard of the International Organization for Standardization (ISO25178 [15]). Both methods have proved to be highly effective in determining the diet of both extant and extinct species of fish [14, 16], stem mammals [17], primates [12, 18, 19], carnivores [20–22], bats [23], and hoofed mammals including suids [13, 24–29]. In particular, Merceron et al. [28, 29] reported the effectiveness of dental microwear texture analysis for detecting dietary differences within the same species, which would also be efficient in detecting the raising conditions of the domestic animals.

In this study, we tested whether STA with ISO parameters can detect any difference between wild and stall-fed populations of *S*. *scrofa*. In addition, we tested whether these parameters differ between subspecies of wild *S*. *scrofa* populations with distinct body sizes. Furthermore, we tested whether these parameters differ between the molars of *S*. *scrofa* to check the applicability of the method to an isolated molar regardless of its tooth position. If the parameters differ between domesticated and wild *S*. *scrofa* but not between wild populations, these parameters would be indicated to reflect the feeding ecology of *S*. *scrofa* regardless of subspecies or body size, and confirm the utility of STA for assessing archaeological remains to distinguish domesticated *S*. *scrofa* from wild *S*. *scrofa*.

## Materials and methods

### Ethics statement

Tooth specimens are stored in The University Museum, The University of Tokyo (UMUT), and the National Museum of Nature and Science, Tokyo (NMNS), or deposited in the Daté City Institute of Funkawan Culture (DCIFC). They were hunted in the 1970s and 1980s with the permission of the Ministry of the Environment, Japan, or slaughtered as domestic animals. Permission was obtained from all museums and institutions to access the specimens for scientific purposes. Hence, no animals were sacrificed for this study.

### Specimens

This study analyzed two wild populations and one stall-fed population of *S*. *scrofa* (Table 1). The wild populations were classified as Wild (L) or Wild (S). All individuals (n = 13) in the Wild (L) group were the Japanese wild boar *S*. *s*. *leucomystax*, which inhabits deciduous broad-leaved forests of the Tanba region, Hyogo Prefecture, western Honshu, Japan. Asahi [30] reported the stomach contents of boars from Hyogo, Kyoto, and Osaka prefectures collected during the hunting season of 1970 (i.e., from November 1970 to February 1971). The leaves, stems, and roots of monocots, such as Gramineae, showed the highest proportion among the stomach contents. Rhizomes (e.g., potato, arrowroot, yam, and sweet potato) and fruits and seeds such as beans and hard nuts were also identified. In addition, 30% of the studied stomachs contained animal matter such as earthworms and insects. In general, fibrous tissues occupied a high proportion of the stomach contents in both volume and weight. Asahi [30] assumed that these fibrous tissues were digested bark and roots. However, in some individuals, bark, roots, and rhizomes were the main stomach contents. The amount of animal matters was considerably lower, although it was frequently observed. In summary, *S*. *s*. *leucomystax* mainly rely on roots, barks, and rhizomes in winter.

**Table 1:** List of specimens.

No.	Tooth Position	NMP^a^ (%)	Facet	Category	Age^b^	Sex	Coll. Date	Institution
No.75 TANBA	M1	0.08	facet 3	Wild (L)	19 to 20 months	female	1970 to 1972	UMUT
No.88 TANBA	M1	0.03	facet 3	Wild (L)	19 to 20 months	male	1970 to 1972	UMUT
No.145 TANBA	M1	0.01	facet 3	Wild (L)	43 to 44 months	female	1970 to 1972	UMUT
No.148 TANBA	M2	0.00	facet 1	Wild (L)	over 55 months	female	1970 to 1972	UMUT
	M3	0.00	facet 1					
No.149 TANBA	M1	0.06	facet 3	Wild (L)	19 to 20 months	male	1970 to 1972	UMUT
No.151 TANBA	M2	0.03	facet 3	Wild (L)	over 55 months	male	1970 to 1972	UMUT
	M3	0.00	facet 3					
No.154 TANBA	M2	0.26	facet 3	Wild (L)	43 to 44 months	male	1970 to 1972	UMUT
	M3	0.11	facet 3					
No.246 TANBA	M2	0.01	facet 3	Wild (L)	31 to 32 months	male	1970 to 1972	UMUT
No.262 TANBA	M1	0.00	facet 3	Wild (L)	19 to 20 months	female	1970 to 1972	UMUT
No.267 TANBA	M1	0.06	facet 3	Wild (L)	31 to 32 months	male	1970 to 1972	UMUT
No.282 TANBA	M2	0.18	facet 1	Wild (L)	43 to 44 months	female	1970 to 1972	UMUT
No.295 TANBA	M2	0.04	facet 3	Wild (L)	43 to 44 months	male	1970 to 1972	UMUT
No.299 TANBA	M2	0.00	facet 3	Wild (L)	31 to 32 months	male	1970 to 1972	UMUT
M31139	M1	3.55	facet 3	Wild (S)	31 to 32 months	male	1980 to 1985	NMNS
	M2	1.74	facet 3					
M31142	M1	1.27	between facet 1 and 3	Wild (S)	43 to 44 months	male	1980 to 1985	NMNS
	M2	0.03	facet 3					
	M3	0.01	facet 3					
M31143	M1	1.46	facet 3	Wild (S)	19 to 20 months	female	1980 to 1985	NMNS
M31148	M1	0.86	facet 3	Wild (S)	19 to 20 months	female	1980 to 1985	NMNS
M31153	M1	0.11	facet 7	Wild (S)	43 to 44 months	male	1980 to 1985	NMNS
	M2	0.50	facet 1					
M31155	M1	0.16	facet 5	Wild (S)	19 to 20 months	female	1980 to 1985	NMNS
M31156	M2	0.76	facet 3	Wild (S)	over 55 months	female	1980 to 1985	NMNS
	M3	0.81	facet 3					
M31158	M1	0.01	facet 3	Wild (S)	19 to 20 months	female	1980 to 1985	NMNS
M31160	M1	0.04	facet 3	Wild (S)	19 to 20 months	female	1980 to 1985	NMNS
ISHII 1	M1	1.71	facet 3	Stall-Fed	19 to 20 months	male	1989	DCIFC
ISHII 4	M1	3.09	between facet 1 and 3	Stall-Fed	19 to 20 months	unknown	unknown	DCIFC
ISHII 6	M1	0.84	facet 3	Stall-Fed	19 to 20 months	female	unknown	DCIFC
ISHII 14	M2	1.27	facet 3	Stall-Fed	19 to 20 months	unknown	1992	DCIFC
ISHII 15	M1	0.77	facet 3	Stall-Fed	19 to 20 months	unknown	1992	DCIFC
ISHII 16	M1	2.98	facet 3	Stall-Fed	19 to 20 months	unknown	1993	DCIFC
ISHII 18	M1	0.12	facet 3	Stall-Fed	19 to 20 months	unknown	1993	DCIFC
ISHII 19	M1	2.47	facet 3	Stall-Fed	19 to 20 months	unknown	1993	DCIFC
ISHII 20	M1	0.15	facet 3	Stall-Fed	19 to 20 months	unknown	1993	DCIFC
	M2	0.31	facet 3					
ISHII 23	M2	5.67	facet 1	Stall-Fed	19 to 20 months	unknown	unknown	DCIFC
ISHII 29	M1	1.52	facet 3	Stall-Fed	19 to 20 months	unknown	unknown	DCIFC
ISHII 2003	M1	0.04	facet 3	Stall-Fed	19 to 20 months	unknown	2003	DCIFC
	M2	0.00	facet 3					

a. NMP: percentage of non-measured points (i.e., replaced points after noise removal).

b. Age of each individual was estimated following Hayashi et al. [34].

All individuals (n = 9) of the Wild (S) group were the Ryukyu wild boar *S*. *s*. *riukiuanus* hunted in the evergreen forest of Iriomote-jima Island, located in the Ryukyu Archipelago, in the south-western part of Japan, although the season in which they were hunted was unknown. Ishigaki et al. [31] reported annual dietary contents of the Iriomote-jima boar based on interviews with hunters and investigation of bite marks left on plants. They reported that *S*. *s*. *riukiuanus* frequently consumed acorns and other fruits from September to the following April. From June to September, the boars often damaged agricultural crops and fruits (e.g., pineapples, bananas, mandarin oranges, and rhizomes). They consumed bamboo shoots and the fruits of evergreen trees from spring to early summer, and the fruits of evergreen shrubs from summer to early autumn. Plant roots were consumed all year round. The hard nuts (i.e., acorns) of evergreen trees were the dietary resource most favored by the boars. Other plant parts, such as fruits, roots, leaves, and the soft tissues of stems, were also reported as included in the diet of the boars. The boars also consumed insects, crabs, earthworms, and snakes. A stable carbon isotope ratio in the dental enamel of the individuals of the Wild (S) population was in the range of those of C_3_ plant feeders (‒14.0‰ [32]). The ranges of body weight of *S*. *s*. *leucomystax* and *S*. *s*. *riukiuanus* are 50 to 150 kg and 40 to 50 kg, respectively [33].

All individuals (n = 12) of the stall-fed population mainly fed on corn in a concrete-floored stall and were slaughtered at 18 months old. They were Japanese wild boars and were thus taxonomically and morphologically identical to the Wild (L) population.

### Data acquisition

**Fig 1:**
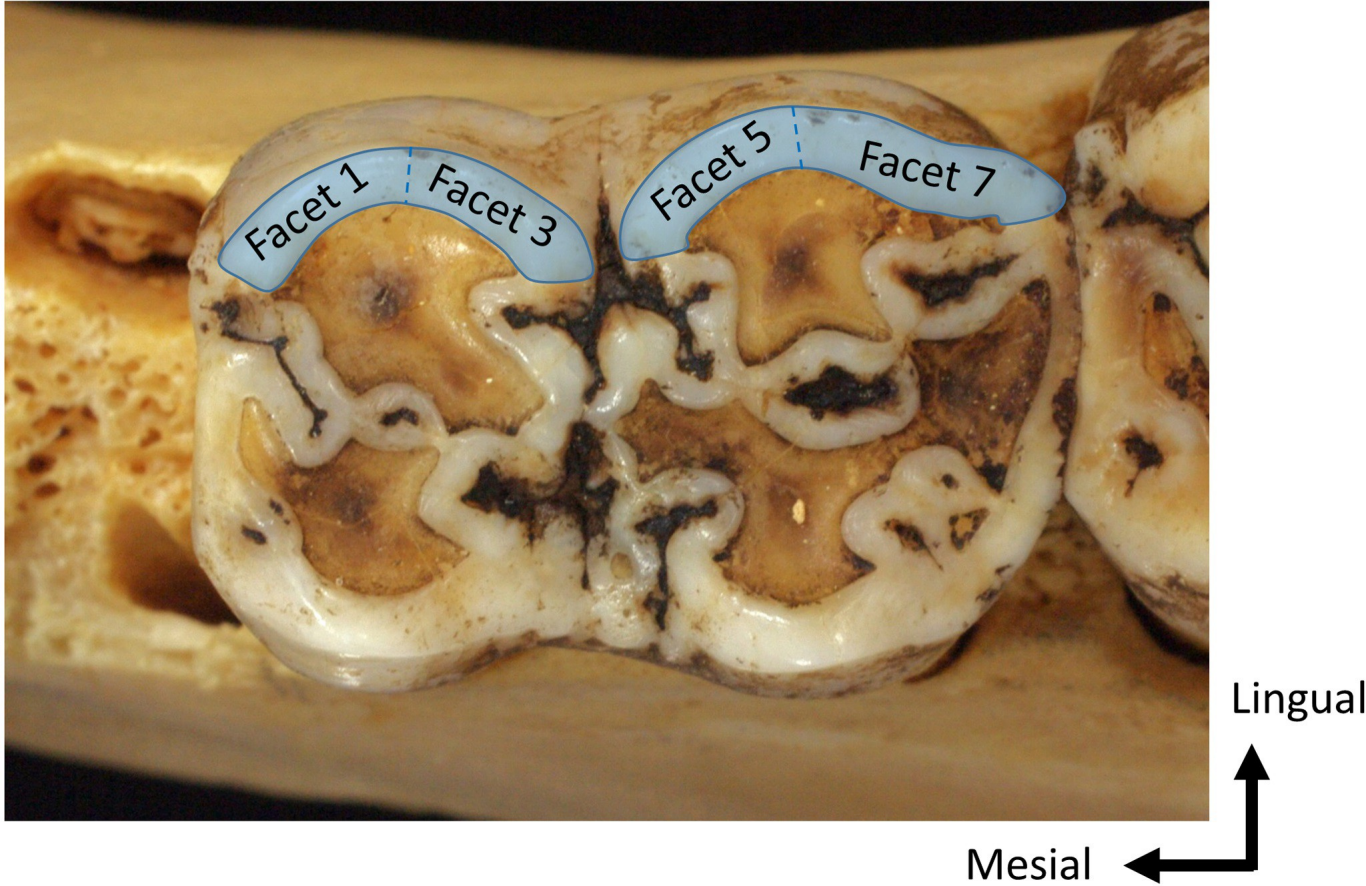
Occlusal enamel facets examined in surface texture analysis. This study generally examined Facet 3, as shown in the figure. In cases where Facet 3 was broken or extremely dirty, one of the facets highlighted in this figure was examined. The facet examined for each specimen is shown in Table 1.

We examined the lingual side of occlusal enamel facets, which occlude during the chewing-shearing phase. We generally examined the surface texture on Facet 3, as shown in Fig 1. In cases Facet 3 was broken or extremely dirty, one of the occlusal enamel facets on the lingual side was examined. This study analyzed specimens with attritional facets, where the tooth enamel of the occlusal plane was not worn out, corresponding to the Individual Dental Age Stages 2 and 3 [35].

After the surfaces of the lower first (M1), second (M2), and third molars (M3) were washed with 100% acetone, tooth surfaces were molded using high-resolution silicone (Affinis light body, Coltene Co, Switzerland). The molds were scanned using a confocal laser microscope (VK-9700, Keyence Co, Japan) with a 100x objective super long-distance lens (numerical aperture = 0.95). The microscope had a photomultiplier tube with a 1024×768 pixel frame. The laser wavelength was 408 nm, and the scan pitches were 0.137 μm/pixel for x- and y- axes, with a vertical resolution of 1 nm. Therefore, the field of view was 140×105 μm. We scanned four adjacent fields, two by two along the x- and y- axes, and combined them into one large field by using VK Assembler software (Keyence Co, Japan). ISO parameters were calculated from these combined fields by using surface roughness software (Mountains Map 7 ver. 7. 4. 8226, Digital Surf Co, France).

Because the molds were mirror images of the real tooth surfaces, the coordinates were mirrored in the x- and z- axes. The surface data were leveled to remove the inclination of the mold. We used a robust Gaussian filter (cut-off scale, 0.8μm) as an S-filter to remove measurement noise, and then applied the form removal function of Mountains Map 7 software (a second order polynomial function) as an F-operation to remove large-scale curvatures of the mold surface. Subsequently, following Arman et al. [36], the features with a slope >80° were considered as noise points and were replaced with the mean of the neighboring points. Percentages of replaced points were calculated for each scan. After these preparations, ISO 25178 parameters were calculated for the 3D coordinates of each mold. A specimen showing over 5% of replaced points (i.e., ISHII 23, Table 1) was excluded from following analyses. As the results of primary comparisons, the data patterns of each ISO parameter among the populations of the same dental age or facet (Facet 3) were similar to the patterns for different ages or facets. Therefore, we considered the effect of a difference in age and/or facet to be negligible.

### Data analysis

To test the applicability of STA against an isolated tooth in later tests, we first tested whether the ISO parameters varied between M1 and M2 (five pairs), or M2 and M3 (five pairs), using matched pairwise comparison (i.e., Wilcoxon signed-rank test).

To examine the sensitivity of the ISO parameters against the feeding ecology of *S*. *scrofa*, each parameter was statistically compared among the populations with Holm adjustment [37]. We performed pairwise comparisons between populations using parametric or non-parametric methods depending on the distribution and variance of each ISO parameter. Before comparisons, the Shapiro Wilk normality test and the Bartlett test of homogeneity of variances were performed to determine which statistical methods were most appropriate. We performed one-way analysis of variance (ANOVA) on the parameters with normal distribution and homogeneity of variance, as well as on the parameters with homogeneity of variance after log transformation. When the data were distributed normally but variances were not homogeneous, a Welch ANOVA was performed. For the ISO parameters that were not normally distributed even after log transformation, the Wilcoxon rank sum test was applied as a non-parametric alternative. The comparisons were performed based on three datasets; 1) M1 only (n = 24), 2) M2 only (n = 14), and 3) either M1 or M2 (n = 33) to increase the sample size. In the last dataset, when several teeth were available for the same individual, we used ISO parameters of the tooth that erupted earlier. Therefore, only one specimen was used for each individual in any case. Statistical tests were performed using R x64 ver. 3.3.3 [38].

## Results

Among the 35 ISO 25178 parameters examined, Sda (average area of dales connected to the edge at a defined height; see S1 Table for basic statistics of the parameters) differed significantly between M1 and M2 (Table 2). Average scores of Sda were 317.93 μm^2^ for M1 and 559.04 μm^2^ for M2.

**Table 2:** Wilcoxon signed-rank test results for differences in ISO25178 parameters between tooth positions in the same individuals.

Parameter	M1 vs. M2^a^	M2 vs. M3^b^
Sq	0.500	0.125
Ssk	0.686	0.125
Sku	0.500	0.625
Sp	0.500	0.188
Sv	0.893	0.313
Sz	0.500	0.313
Sa	0.500	0.125
Smr	0.893	0.313
Smc	0.500	0.125
Sxp	0.686	0.063
Sal	0.345	0.625
Str	0.225	0.063
Std	0.345	1.000
Sdq	0.345	0.625
Sdr	0.345	0.625
Vm	0.345	0.063
Vv	0.500	0.125
Vmp	0.345	0.063
Vmc	0.686	0.313
Vvc	0.500	0.125
Vvv	0.893	0.063
Spd	0.225	0.438
Spc	0.500	0.625
S10z	0.893	1.000
S5p	0.686	0.063
S5v	0.893	0.813
Sda	**0.043**	1.000
Sha	0.080	0.813
Sdv	0.893	1.000
Shv	0.080	0.438
Sk	0.345	0.125
Spk	0.225	0.188
Svk	0.500	0.813
Smr1	0.345	0.060
Smr2	0.500	0.625

Boldface font indicates *p*<0.05.

a. Comparison of M31139, M31142, M31153, ISHII20, and ISHII2003.

b. Comparison of No.148 TANBA, No.151 TANBA, No.154 TANBA, M31142, and M31156.

Pairwise comparisons among the populations that used either M1 or M2 were performed using all ISO parameters except Sda, which differed significantly between M1 and M2. Consequently, the stall-fed and Wild (S) populations differed significantly from the Wild (L) population in 20 and six parameters, respectively (Table 3).

**Table 3:** Pairwise comparisons of *Sus* populations using either M1 or M2 with Holm adjustment for differences in ISO25178 parameters.

Parameter	Description	Population		
			Stall-Fed	Wild (L)
Sq^a^	Root mean square height	Wild (L)	**0.002**	-
		Wild (S)	0.175	0.175
Sv^b^	Maximum pit height	Wild (L)	**0.001**	-
		Wild (S)	0.116	0.116
Sz^a^	Maximum height	Wild (L)	**0.003**	-
		Wild (S)	0.173	0.173
Sa^a^	Arithmetical mean height	Wild (L)	**0.003**	-
		Wild (S)	0.150	0.150
Smc^a^	Height at a given material ratio^c^ p (p = 10%)	Wild (L)	**0.003**	-
		Wild (S)	0.202	0.202
Sxp^b^	Difference in height between q% and p% material ratio^c^ (p = 50%, q = 97.5%)	Wild (L)	**0.002**	-
		Wild (S)	0.149	0.149
Sdq^b^	Root mean square gradient	Wild (L)	**<0.001**	-
		Wild (S)	0.235	**0.018**
Sdr^b^	Developed interfacial area ratio	Wild (L)	**0.001**	-
		Wild (S)	0.311	**0.031**
Vv^a^	Void volume at a given material ratio^c^ p (p = 10%)	Wild (L)	**0.003**	-
		Wild (S)	0.208	0.208
Vmc^b^	Material volume in the core or kernel, between two material ratios^c^ p and q (p = 10%, q = 80%), calculated in the zone between c1 and c2	Wild (L)	**0.004**	-
		Wild (S)	0.203	0.127
Vvc^a^	Void volume in the core or kernel, between two material ratios^c^ p and q (p = 10%, q = 80%), calculated in the zone between c1 and c2	Wild (L)	**0.004**	-
		Wild (S)	0.225	0.225
Vvv^b^	Void volume in the valleys, between a material ratio^c^ p and q (p = 80%, q = 100%), calculated in the zone below c2	Wild (L)	**0.001**	-
		Wild (S)	0.143	0.143
Spc^a^	Arithmetic mean peak curvature	Wild (L)	**<0.001**	-
		Wild (S)	0.113	**0.019**
S10z^b^	Ten-point height	Wild (L)	**<0.001**	-
		Wild (S)	0.107	0.069
S5p^b^	Five-point peak height	Wild (L)	**0.003**	-
		Wild (S)	0.300	0.059
S5v^b^	Five-point pit height	Wild (L)	**0.003**	-
		Wild (S)	0.176	0.176
Sha^b^	Average area of hills connected to the edge at height c (c: height at 50% material ratio)	Wild (L)	**0.025**	-
		Wild (S)	0.805	0.051
Sk^b^	Distance between the highest and lowest level of the core surface	Wild (L)	**<0.001**	-
		Wild (S)	0.281	**0.021**
Spk^b^	Average height of the protruding peaks above the core surface	Wild (L)	**<0.001**	-
		Wild (S)	0.206	**0.028**
Svk^b^	Average height of the protruding dales below the core surface	Wild (L)	**<0.001**	-
		Wild (S)	0.113	**0.019**

Boldface font indicates *p*<0.05

a. One-way analysis of variance.

b. Wilcoxon rank sum test.

c. Material ratio: the ratio of the surface area over a given height c to the entire surface area.

**Fig 2:**
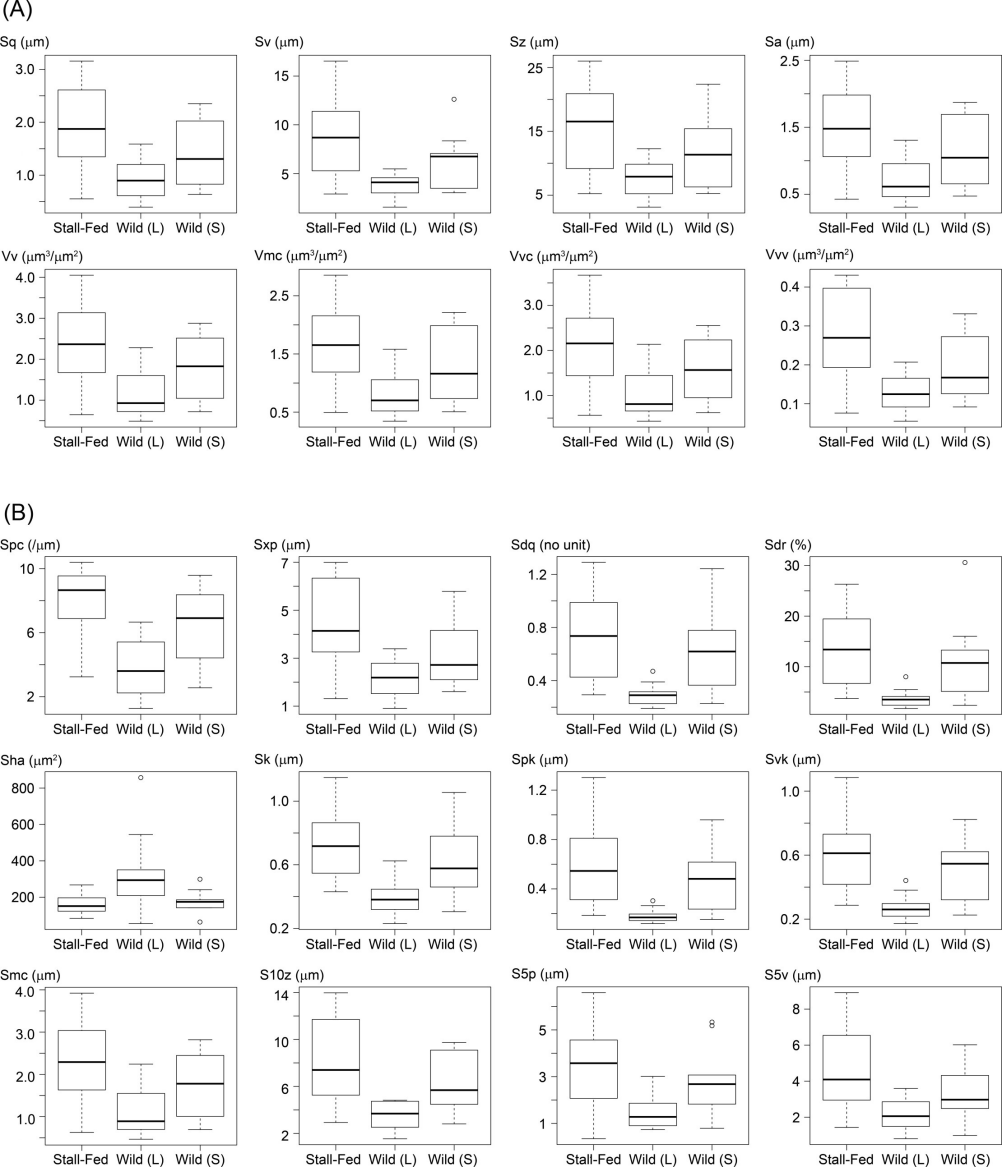
Boxplots of 20 ISO parameters that differed significantly among the studied populations using either M1 or M2. (A) Parameters related to height and volume. **(B) Parameters related to other textures.** All parameters differed significantly between the stall-fed and Wild (L) populations and six parameters differed significantly between the two wild populations. No parameter differed significantly between the stall-fed and Wild (S) populations. The results of statistical comparisons and the description of each ISO parameter are presented in Table 4. The box encloses the 25th and 75th percentiles, with the horizontal line representing the median. Outliers (open circles) are 1.5 times greater than the extreme end of the interquartile range. When ISO parameters were obtained from several teeth in the same individual, the parameters of the tooth that erupted earlier were used.

Among the three populations, the stall-fed or Wild (L) populations showed the highest or lowest average values and the value for the Wild (S) population was between the other two populations for all 20 parameters, which differed significantly between populations (Table 3 and Fig 2A and 2B). The value of the height parameters of the surface texture (i.e., Sq, Sv, Sz, and Sa) were significantly larger in the stall-fed population, indicating higher hill peaks and deeper dales on the tooth surface compared to those in the Wild (L) population (Fig 2A). The parameters related to volume were also larger in the stall-fed population. Higher values of Vv, Vvc, and Vvv indicated that the valley structures were larger in volume, and higher values of Vm, Vmc, and Vmp indicated larger hill volume on the tooth surface of the stall-fed population compared with the Wild (L) population (Fig 2A). Six parameters (Sdq, Sdr, Spc, Sk, Svk, and Spk) differed significantly between the Wild (L) population and the other two populations (Fig 2B). Larger values in Sdq and Sdr indicated steeper hills and dales, and higher values of Spc and Spk indicated more pointed, angled peaks in the Wild (S) and the stall-fed populations compared with the Wild (L) population. Conversely, neither height nor volume parameters differed significantly between the two wild populations.

When pairwise comparisons among the populations were performed separately for M1 and M2, the results were similar to those using both M1 and M2, although the number of significant parameters was much smaller for the M2-only dataset (Table 4). Again, the parameters that differed significantly between M1 and M2 (i.e., Sda) did not differed significantly among the populations. Comparisons using M1 revealed that 20 parameters differed significantly between the Wild (L) and the stall-fed populations, whereas only one parameter differed significantly between the Wild (L) and the Wild (S) populations (Table 4). In addition, Std (the direction of texture, S1 Table) differed significantly between the Wild (S) and stall-fed populations. Comparisons using M2 revealed that three parameters differed significantly between the Wild (L) and stall-fed populations, whereas no significant difference was found between the Wild (L) and Wild (S) populations (Table 4).

**Table 4:** Pairwise comparisons among the studied *Sus* populations with Holm adjustment for differences in ISO25178 parameters using lower first or second molars.

Tooth Position	Parameter	Population		
			Stall-Fed	Wild (L)
M1	Sq^a^	Wild (L)	**0.015**	-
		Wild (S)	0.193	0.193
	Sv^a^	Wild (L)	**0.033**	-
		Wild (S)	0.284	0.284
	Sz^a^	Wild (L)	**0.040**	-
		Wild (S)	0.280	0.280
	Sa^a^	Wild (L)	**0.014**	-
		Wild (S)	0.191	0.191
	Sxp^a^	Wild (L)	**0.027**	-
		Wild (S)	0.194	0.194
	Vv^a^	Wild (L)	**0.008**	-
		Wild (S)	0.149	0.149
	Vmc^a^	Wild (L)	**0.017**	-
		Wild (S)	0.187	0.150
	Vvc^a^	Wild (L)	**0.007**	-
		Wild (S)	0.155	0.155
	Vvv^a^	Wild (L)	**0.032**	-
		Wild (S)	0.155	0.252
	Spc^a^	Wild (L)	**0.007**	-
		Wild (S)	0.157	0.089
	S10z^a^	Wild (L)	**0.039**	-
		Wild (S)	0.237	0.237
	S5v^a^	Wild (L)	**0.035**	-
		Wild (S)	0.296	0.296
	Sk^a^	Wild (L)	**0.003**	-
		Wild (S)	0.095	0.069
	Std^b^	Wild (L)	**0.035**	-
		Wild (S)	**0.027**	0.943
	Smc^c^	Wild (L)	**0.008**	-
		Wild (S)	0.151	0.151
	Sdq^c^	Wild (L)	**0.013**	-
		Wild (S)	0.169	0.135
	Sdr^c^	Wild (L)	**0.024**	-
		Wild (S)	0.211	0.179
	Sha^c^	Wild (L)	**0.035**	-
		Wild (S)	0.773	**0.045**
	Spk^c^	Wild (L)	**0.012**	-
		Wild (S)	0.159	0.943
	Svk^c^	Wild (L)	**0.004**	-
		Wild (S)	0.113	0.113
M2	Vvv^a^	Wild (L)	**0.038**	-
		Wild (S)	0.392	0.392
	S5v^a^	Wild (L)	**0.044**	-
		Wild (S)	0.258	0.362
	S10z^c^	Wild (L)	**0.034**	-
		Wild (S)	0.357	0.357

Boldface font indicates *p*<0.05

a. One-way analysis of variance (ANOVA) performed after log transformation.

b. ANOVA performed after log transformation.

c. Wilcoxon rank sum test.

## Discussion

One surface roughness parameter (Sda) differed significantly between M1 and M2. The results suggested that some ISO parameters should not be applied in STA regardless of tooth position. Considering the small number of specimens used in the matched pairwise comparison, we expect ISO parameters to differ when we increase the sample size. When performing STA of archaeological remains, therefore, tooth position should be standardized if it can be identified. Considering the applicability for domesticated animals, which are often slaughtered before all permanent teeth have erupted, M1 is an ideal position for zooarchaeological studies because it erupts earlier than other permanent teeth.

Sda, however, did not differ significantly between M2 and M3. Other surface roughness parameters (e.g., Sa) also showed no significant differences between the molar positions. To determine the effect of mixing information from different molar positions, we performed three comparisons of ISO parameters other than Sda among the populations, using datasets that included 1) M1 only, 2) M2 only, and 3) either M1 or M2. Surprisingly, despite reducing the sample size from 34 (either M1 or M2) to 24 (M1 only), we found an equal number of significant parameters. All comparisons detected significant differences between the Wild (L) and stall-fed populations, and in fewer parameters between the Wild (L) and Wild (S) populations. Comparisons of ISO parameters revealed significant differences between the stall-fed and Wild (L) populations, although they were similar to each other in body size, shape of skull, and belong to the same subspecies (Table 3). These results are unsurprising because their feeding ecology differs. Foraging of artificial hay from a concrete floor in the stall-fed population should have caused less abrasive wear than rooting rhizome on the forest floor in the Wild (L) population. In addition to the difference in the physical properties of their diets, the difference in the amount of soil and dust contamination during foraging may also have affected the microwear characters. As described by Lucas et al. [39], exogenous grids create distinctive scars on tooth enamel surface. Hoffmann et al. [40] also reported a significant positive correlation between grid size and the frequency of pits on tooth surface enamel. Although Merceron et al. [41] reported that the amount of dust had no significant effect on the enamel textures of sheep, the amount and frequency of soil contamination differed remarkably between sheep and boars. Rooting should affect tooth surface texture [3]. Regardless, both changes of diet and/or foraging behavior resulted in different tooth surface textures in STA. Therefore, we conclude that dietary segregation from the natural environment by domestication caused tooth surface texture differences that can be detected by ISO parameters.

Conversely, no significant difference was observed between the stall-fed and Wild (S) populations in the present study, but the two wild populations differed significantly in six parameters in comparisons using either M1 or M2 (Table 3). These results were surprising, because we expected that the rooting behavior of the wild populations will cause significant deviation in ISO parameters from that of the stall-fed population. The Wild (S) population differed from the others in feeding habits, body size, and phylogenetic background. Concluding which factors were the most crucial in forming the tooth surface texture is difficult. The results showed that STA was not always a powerful tool for identifying domesticated individuals. Previous studies analyzing several species with different body sizes [13, 26] have suggested that feeding ecology (e.g., diet, foraging behavior, exogenous grits, vegetation, and climate) is the key factor in forming characteristics of tooth surface texture. Therefore, we also require detailed feeding ecological information of the individuals in the Wild (S) population to reach a conclusion on this issue.

**Fig 3:**
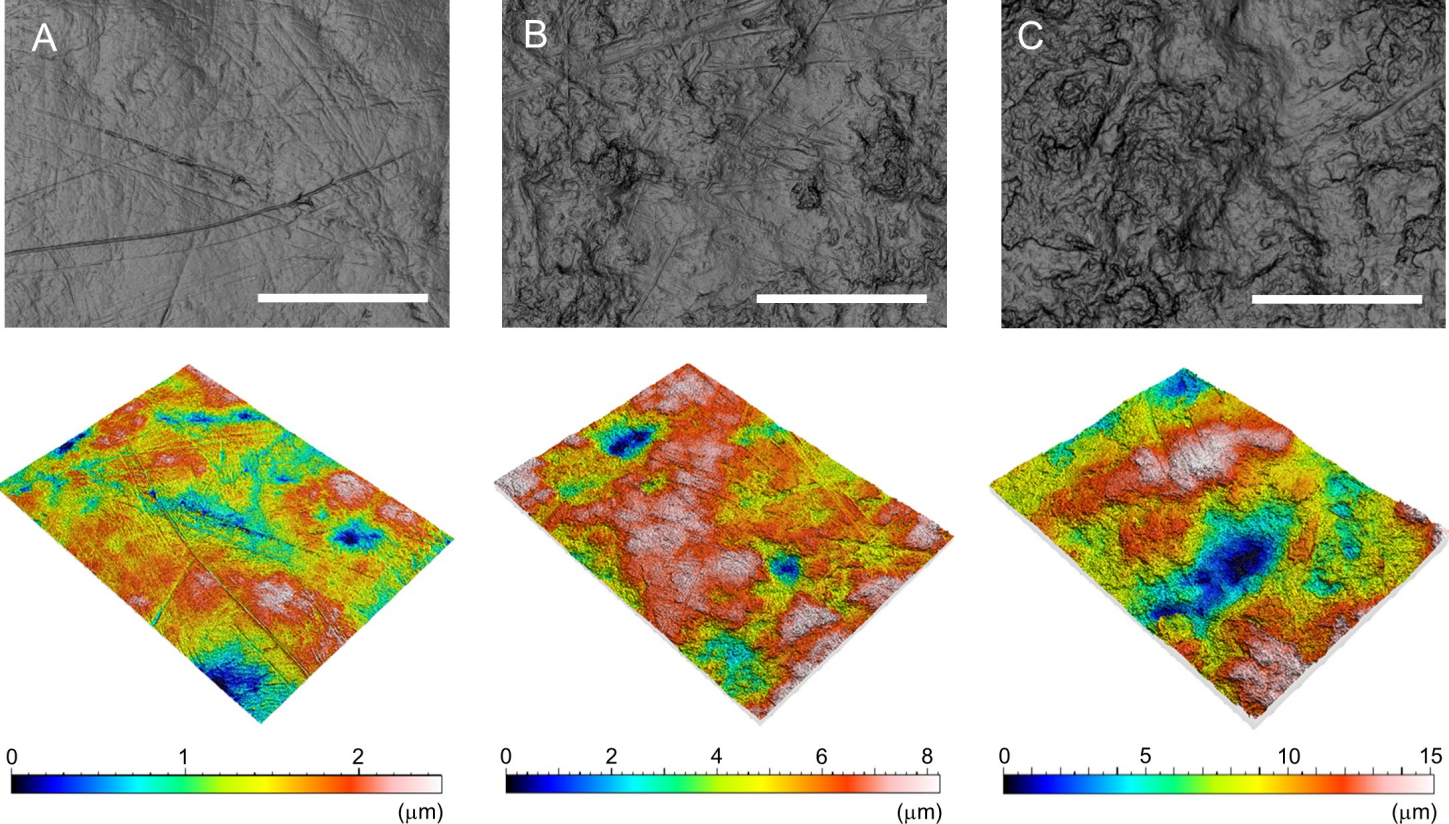
Representative images of microwear on the tooth surface of the studied populations, reconstructed from 3D coordinates. The 3D images (lower images) are mirrored from the 2D images (upper images). (A) Wild (L), (B) Wild (S), (C) Stall-fed. Scale bar = 0.1 mm.

Ward and Mainland [3] reported that the microwear of free-range boars in large grassland paddocks were characterized by a high frequency of scratches, which was an indicator of shearing foods with abrasive matter such as soil contaminated during rooting. By contrast, the tooth surfaces of stall-fed pigs were primarily dominated by pits or gouges, which are typical microwear characteristics, indicative of the crushing of hard objects [42, 43] or the pulping of soft food [44, 45]. Two-dimensional images reconstructed from 3D coordinates taken from our specimens also indicated that the tooth surfaces of the Wild (L) population, which performed rooting in deciduous forests, were dominated by scratches, whereas those of the stall-fed population, which fed on corn hay (i.e., they did not perform rooting), were dominated by pits (Figs 3 and S1, S2, and S3). Nevertheless, the microwear of the Wild (S) population showed more variation; some was dominated by scratches but some was dominated by pitting. The year-round consumption of nuts by the Wild (S) population may have contributed to the similarity of the microwear to that of the stall-fed population (S2 Fig). As mentioned, both the crushing of hard objects and the tooth-to-tooth contact associated with the soft food pulping are known to create pits and gouges, increasing the height and volume of the tooth surface texture. Calandra et al. [43] reported that some primate species consuming mainly soft food showed similar ISO parameter values to those of the other species that rely on hard nuts.

Furthermore, we must consider the effect of human encroachment on the habitat of the Wild (S) population. Ishigaki [31] reported that the Ryukyu wild boar forages on agricultural fruits and crops, particularly from June to September, in addition to hard nuts such as acorns in other seasons. In the case of individuals in the Wild (S) population, which also often invade cultivation areas, it is unsurprising that their tooth surface textures were similar to those of reared pigs (i.e., artificially fed on crops). Winkler et al. [46] also reported that the tooth surface textures of modern small mammals were affected by forest cultivation. Some of the Wild (S) population may have consumed agricultural crops before being hunted, which resulted in the surface texture of their teeth being similar to that of the stall-fed population. The zooarchaeological specimens probably also contained some free-range individual inhabiting a cultivation area after harvest season. If the similarity between the Wild (S) and the stall-fed populations was due to the consumption of agricultural crops, STA might be able to classify those archaeological individuals similarly to the Wild (S) population indicating they were not fully domesticated, but rather at a transitional stage from wild to reared.

However, if the consumption of nuts caused the similarity between the Wild (S) and the stall-fed populations, biochemical analyses for carbides or fatty acids left on artificial remains (e.g., pottery) co-excavated with *Sus*, and/or application of both STA and stable isotope analysis of the same individuals will be necessary to propose a reliable hypothesis of the feeding ecology based on zooarchaeological *Sus* remains. Therefore, in addition to studies using modern specimens with a known diet, combination with other methods is necessary to reconstruct the sequential dietary transition corresponding to the degree of domestication at the individual level. The non-destructive nature of STA is a remarkable advantage of this approach.

The present study showed the caution required in applying STA with the ISO parameters for archaeological *Sus* remains to categorize them as wild or domesticated. The comparison between the stall-fed and Wild (L) populations, in which diet was the primary variable that differed, suggested that the differences were due to the different feeding ecology detected in the ISO parameters. However, the wild *S*. *scrofa* that fed on hard objects and/or agricultural resources could be misjudged as stall-fed individuals based on STA. In other words, wild boars that feed on tough objects such as leaves, like the Wild (L) population in this study, can be distinguished from domesticated pigs, but wild boars that feed on hard objects may not be distinguishable from domesticated pigs.

## Supporting information

S1 Fig Two dimensional teeth surface images of the individuals of Wild (L) population. Scale bar = 0.1 mm. (A) No.75, (B) No.88, (C) No.145, (D) No.148 M2, (E) No.148 M3, (F) No.149, (G) No.151 M2, (H) No.151 M3, (I) No.154 M2, (J) No.154 M3, (K) No.246, (L) No.262, (M) No.267, (N) No.282, (O) No.295, (P) No.299.
(TIF)

S2 Fig Two dimensional teeth surface images of the individuals of Wild (S) population. Scale bar = 0.1 mm. (A) M31139 M1, (B) M31139 M2, (C) M31142 M1, (D) M31142 M2, (E) M31142 M3, (F) M31143, (G) M31148, (H) M31153 M1, (I) M31153 M2, (J) M31155, (K) M31156 M2, (L) M31156 M3, (M) M31158, (N) M31160.
(TIF)

S3 Fig Two dimensional teeth surface images of the individuals of stall-fed population. Scale bar = 0.1 mm. (A) ISHII 1, (B) ISHII 4, (C) ISHII 6, (D) ISHII 14, (E) ISHII 15, (F) ISHII 16, (G) ISHII 18, (H) ISHII 19 M1, (I) ISHII 20 M2, (J) ISHII 20, (K) ISHII 23, (L) ISHII 29, (M) ISHII 2003 M1, (N) ISHII 2003 M2.
(TIF)

S1 Table All values of ISO25178 parameters. a. Material ratio: the ratio of surface area over given height c to all surface area.
(XLSX)
