# Supplementary figures and images for: Three-dimensional tooth surface texture analysis on stall-fed and wild boars (Sus scrofa)
Source: PLoS One. 2018 Oct 23;13(10):e0204719. doi: 10.1371/journal.pone.0204719 (PMC6198946; doi:10.1371/journal.pone.0204719)

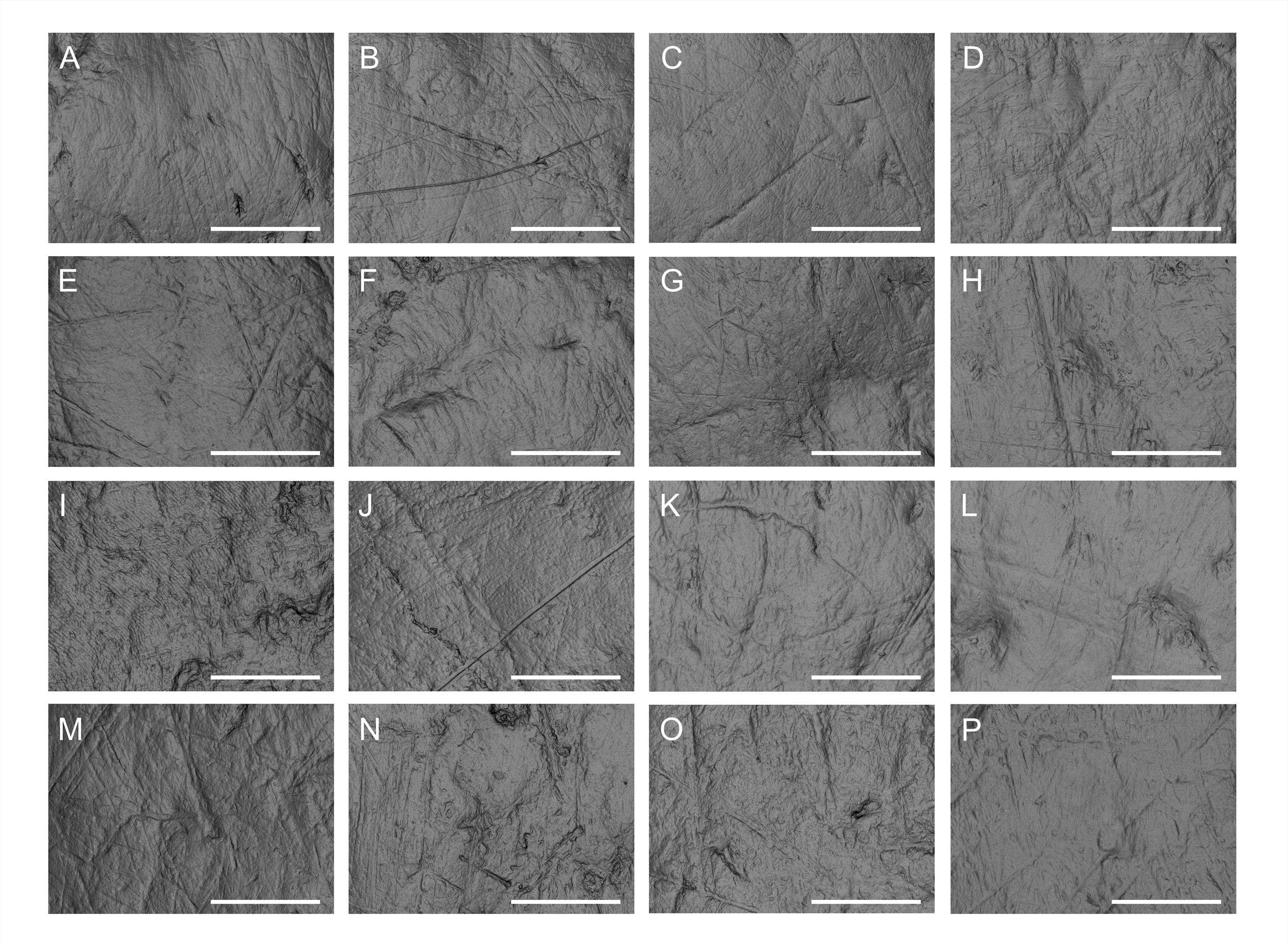

Supplement: S1 Fig — Scale bar = 0.1 mm. (A) No.75, (B) No.88, (C) No.145, (D) No.148 M2, (E) No.148 M3, (F) No.149, (G) No.151 M2, (H) No.151 M3, (I) No.154 M2, (J) No.154 M3, (K) No.246, (L) No.262, (M) No.267, (N) No.282, (O) No.295, (P) No.299. (TIF) [file pone.0204719.s001.tif]

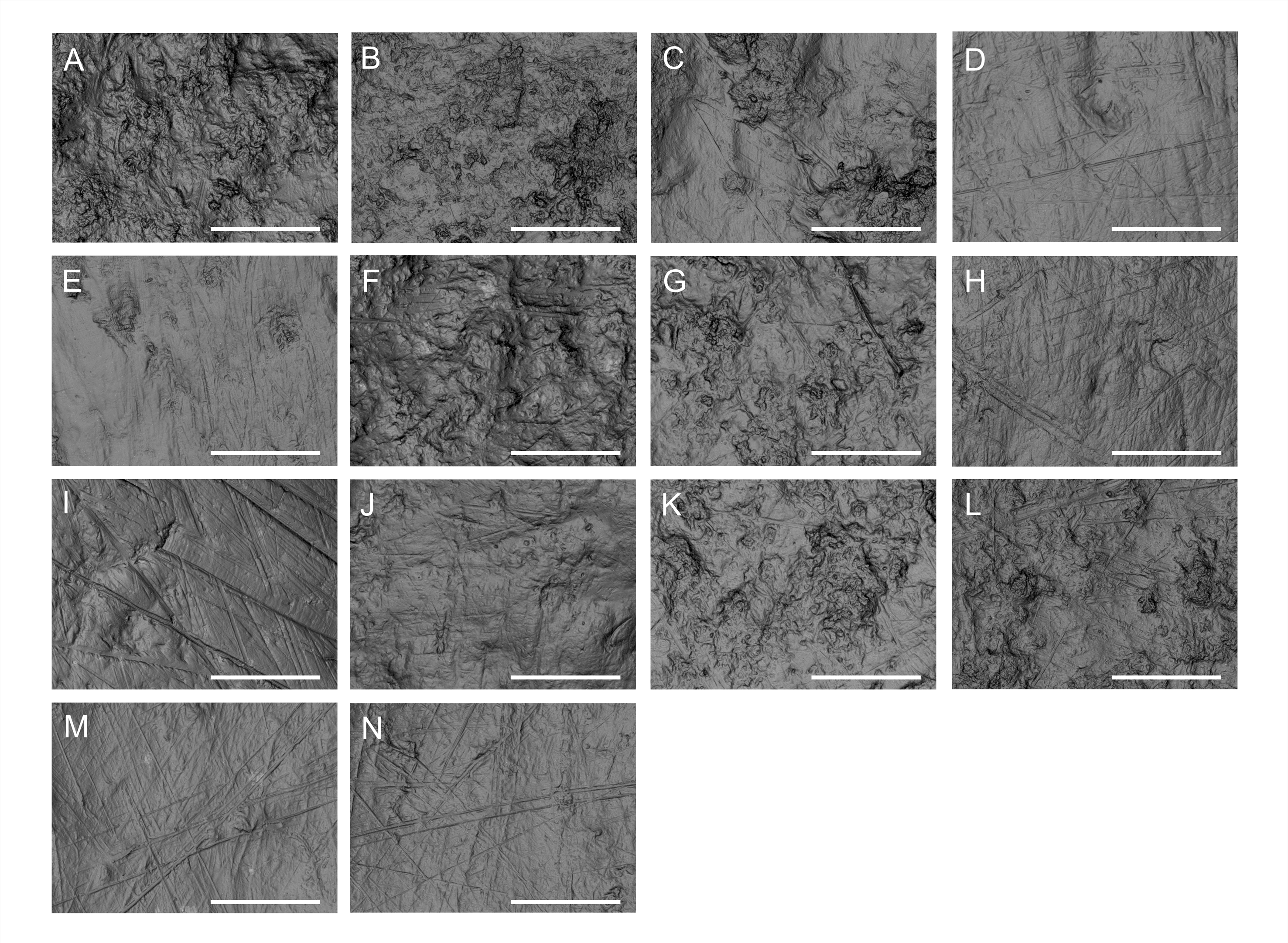

Supplement: S2 Fig — Scale bar = 0.1 mm. (A) M31139 M1, (B) M31139 M2, (C) M31142 M1, (D) M31142 M2, (E) M31142 M3, (F) M31143, (G) M31148, (H) M31153 M1, (I) M31153 M2, (J) M31155, (K) M31156 M2, (L) M31156 M3, (M) M31158, (N) M31160. (TIF) [file pone.0204719.s002.tif]

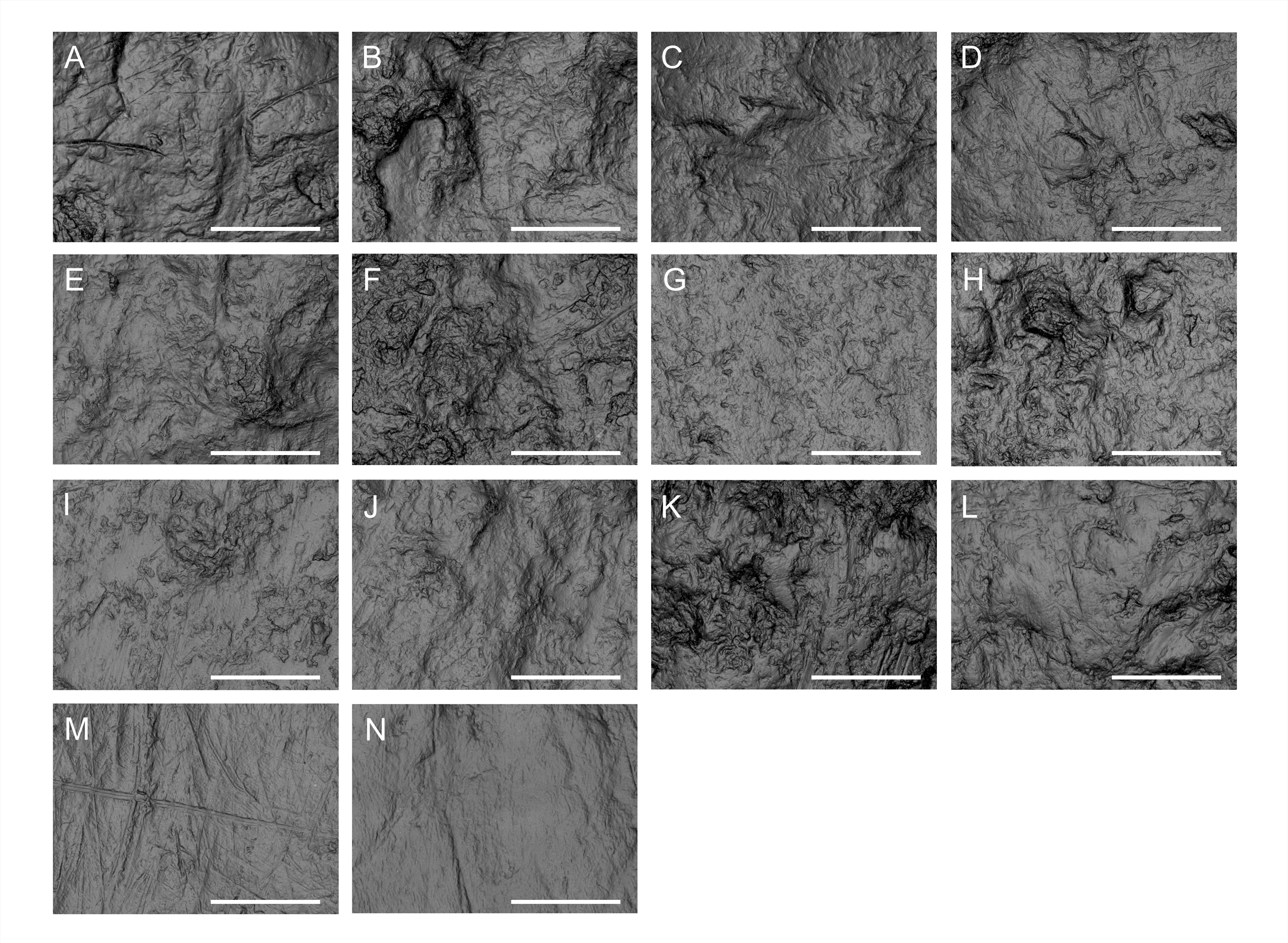

Supplement: S3 Fig — Scale bar = 0.1 mm. (A) ISHII 1, (B) ISHII 4, (C) ISHII 6, (D) ISHII 14, (E) ISHII 15, (F) ISHII 16, (G) ISHII 18, (H) ISHII 19 M1, (I) ISHII 20 M2, (J) ISHII 20, (K) ISHII 23, (L) ISHII 29, (M) ISHII 2003 M1, (N) ISHII 2003 M2. (TIF) [file pone.0204719.s003.tif]
